# Expression of CD74 is increased in neurofibrillary tangles in Alzheimer's disease

**DOI:** 10.1186/1750-1326-3-13

**Published:** 2008-09-11

**Authors:** Kathryn J Bryan, Xiongwei Zhu, Peggy L Harris, George Perry, Rudy J Castellani, Mark A Smith, Gemma Casadesus

**Affiliations:** 1 Department of Pathology, Case Western Reserve University, Cleveland Ohio, USA; 2 College of Sciences, University of Texas at San Antonio, San Antonio, Texas, USA; 3 Department of Pathology, University of Maryland, Baltimore, Maryland, USA; 4 Department of Neurosciences, Case Western Reserve University, Cleveland Ohio, USA

## Abstract

Alzheimer disease (AD) is a chronic neurodegenerative disease that is characterized by progressive memory loss. Pathological markers of AD include neurofibrillary tangles, accumulation of amyloid-β plaques, neuronal loss, and inflammation. The exact events that lead to the neuronal dysfunction and loss are not completely understood. However, pro-inflammatory cytokines, such as interleukin-1β, interleukin-6, and tumor necrosis factor α, are increased in AD, along with gene expression of major histocompatibility complex (MHC) class II molecules and macrophage migration inhibitory factor (MIF). MHC class II molecules are found in microglia of the brain, while MIF is found in both microglia and neurons of the hypothalamus, hippocampus, and cortex. MIF is not only a lymphocyte mediator but also a pituitary factor with endocrine properties and can mediate phosphorylation of the extracellular signal-regulated kinase-1/2 MAP kinases pathway. In this study, we looked at CD74, an integral membrane protein that acts as both a chaperone for MHC class II molecules as well as a receptor binding site for MIF. CD74 was recently found to be increased in microglia in AD cases compared to age-matched controls, but has not been reported in neurons. In our analysis, immunohistochemistry revealed a significant increase in CD74 primarily in neurofibrillary tangles, amyloid-β plaques, and microglia. This is the first finding to our knowledge that CD74 is increased in neurons of AD cases compared to age-matched control cases.

## Background

Alzheimer disease (AD) is a chronic neurodegenerative disease that is pathologically characterized by neurofibrillary tangles (NFT), accumulation of amyloid-β (Aβ), and increased inflammatory markers. However, many lines of evidence suggest that mitogenic signaling, sex steroids, oxidative stress and inflammation may also play a role in the pathogenesis of the disease [1-3]. Increased inflammation has been associated with cognitive decline in AD. Pro-inflammatory markers, such as interleukin-1β (IL-1β), interleukin-6 (IL-6), and tumor necrosis factor α (TNFα) are not only increased in AD patients, but also increase Aβ [4,5]. The accumulation of Aβ then induces a pro-inflammatory response, continuing the cycle.

Recently, Parachikova and colleagues [6] found that there was increased gene expression of the inflammatory molecule, major histocompatibility complex (MHC) II in the hippocampus of AD cases with mild/moderate dementia. MHC class II was found primarily in the microglia and the increase in MHC class II was correlated with cognitive decline based on the mini-mental state exam (MMSE) score. MHC class II is responsible for displaying degraded foreign proteins to the cell surface for the recognition by CD4+ T lymphocytes. CD74, also known as the invariant chain, is known for its role in chaperoning the MHC class II molecules from the endoplasmic reticulum to the cell surface. CD74 blocks the peptide-binding site of MHC class II molecules during transportation from the ER to the cell surface [7].

Interestingly CD74 also mediates binding of the extracellular pro-inflammatory cytokine macrophage migration inhibitory factor (MIF) [8]. MIF is released in response to stress or an inflammatory response. MIF down-regulates the immunosuppressive effects of glucocorticoids and has been found to increase in the brain and cerebrospinal fluid in response to lipopolysaccharide [9]. Furthermore, MIF can be induced to form amyloid fibrils and can bind to Aβ [10,11]. The role of CD74 as an MHC class II chaperone and as a receptor for MIF, both of which contribute to the pathology of AD, lead us to believe that CD74 may play an important role in AD. Yoshiyama determined that CD74 is increased in microglia of AD patients by means of immunohistochemistry, but did not examine neuronal CD74 [12]. Increased MIF mRNA was found in AD patients in neurons [13]. Therefore, the aim of this study is to determine if CD74 is altered in AD patients in both the microglia and neurons.

## Results

### Immunocytochemistry

**Figure 1:**
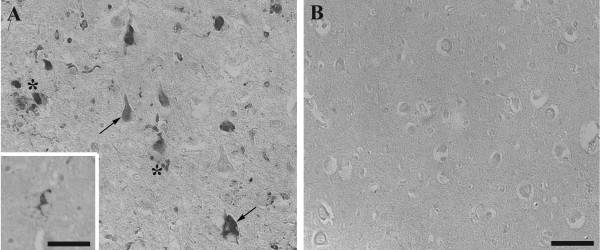
**Immunohistochemistry with the antibody to CD74 in AD (A) and control (B) hippocampus.** CD74 strongly stained neurofibrillary tangles (arrows), amyloid-β plaques (*) and microglia (inset) in the AD cases (n = 6), but not in controls (n = 6). Scale Bar = 50 μm.

CD74 immunocytochemistry demonstrated labeling of microglial processes within the gray matter of the hippocampal pyramidal cell layer and the subiculum, and was most notable within microglial processes in and around senile (neuritic and cored) plaques. This finding is overall consistent with the literature on CD74 in AD brains. In addition, however, we noted strong immunolabeling of pyramidal neurons, both diffusely within the cytoplasm of pathologically normal neurons, as well as co-labeling of neurofibrillary pathology; in particular, neurofibrillary tangles, neuropil threads, and dystrophic neurites within neuritic senile plaques also showed strong immunoreactivity in a distribution similar to phospho-tau immunoreactivity. Labeling of amyloid plaque cores per se and diffuse plaques was not identified, nor was labeling of blood vessels affected by cerebral amyloid angiopathy. No labeling of granulovacuolar degeneration or Hirano bodies was seen. No significant immunolabeling of macroglia (astrocytes, oligodendrocytes) was present. Control tissue showed no immunolabeling, either of neurons, macroglia, or microglia (Figure 1A,B).

### Immunoblotting

**Figure 2:**
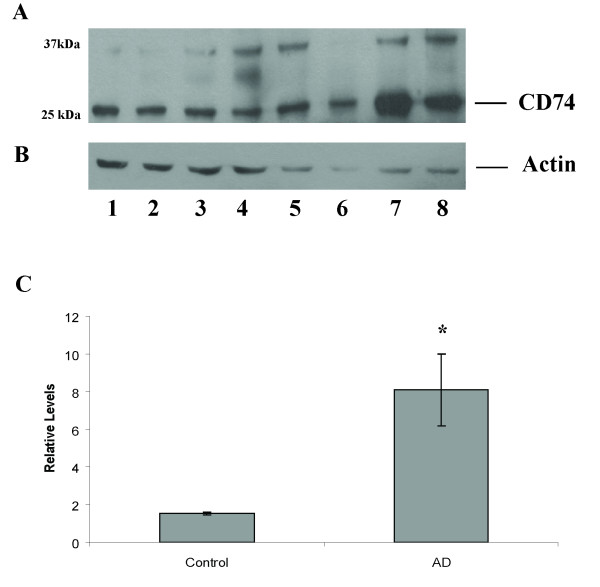
**CD74 protein levels were assessed by Western blotting in four AD cases (lanes 5–8) and four controls (lanes 1–4).** (A) Western blot for CD74. (B) Actin. The relative quantification and statistical analysis shows significantly higher (p =< 0.01) levels of CD74 in AD verses control brain (C).

Immunoblot of brain homogenates prepared from AD and control hippocampus demonstrated protein bands at approximately 30–37 kDa, consistent with reported CD74 isoforms (Figure 2A). Statistical analysis of the 30 kDa band showed a significantly higher level of CD74 in AD compared to control (p =< 0.01) (Figure 2C).

## Discussion

AD is the most common neurodegenerative disorder, but the cause of AD is still unknown. Pro-inflammatory markers IL-1β, IL-6, and TNFα are increased in AD patients as well as the anti-inflammatory marker IL-10 [4,5]. Increases in two additional inflammatory markers, MIF and MHC class II recently have been found in AD cases compared to age-matched controls [6,8].

Immunohistochemistry results (Figure 1A,B) reveal that the MIF receptor CD74 is increased in NFTs and microglia of AD patients compared to age-matched controls. To our knowledge this is the first study to find increased CD74 expression in neurons. Immunoblot analyses confirmed our findings from the immunohistochemistry data in that CD74 was expressed at the correct molecular weight in both AD and control brains (Figure 2). Overall there was a significant increase in CD74 in AD compared to control cases, which may be related to the disease stage, with two AD cases being more severe than the others and likely containing more CD74-containing neurofibrillary pathology. Additional cases will need to be analyized to confirm this. Insoluble fragments were found at the top of the blot, which may be due to the insoluble paired helical filaments that compose NFTs [14]. This study is consistent with previous findings that CD74 is increased in the microglia of AD brain, but sheds light that CD74 is important in NFTs as well.

The fact that CD74 is an extracellular surface receptor for MIF was only recently discovered [8]. MIF has been found in neurons of the hippocampus, hypothalamus, and cortex and is often increased in AD patients compared to controls [10]. CD74 is a high affinity binding protein for MIF and is necessary for MIF-induced activation of the extracellular-regulated-kinase (ERK) 1/2 MAP kinase cascade, cell proliferation, and prostaglandin E_2 _(PGE_2_) production [8]. In this regard, it is of interest to note that the ERK pathway is activated in AD [14-17] and elevated levels of PGE_2 _in AD are likely involved in Aβ production [18,19]. CD74 is mainly known as an MHC class II chaperone, which allows the MHC class II molecules to leave the endoplasmic reticulum and prevents binding of other proteins to the MHC II molecule. Stimulation of CD74 leads to NFκB activation, entry of CD74 stimulated cells into the S phase, elevation of the DNA synthesis, cell division, and leads to cell proliferation and survival [20,21].

There is very little information on the role of CD74 in neurodegenerative diseases. Yoshiyama and colleagues [12] did determine that CD74 was increased in only resting or mildly active microglia of AD patients. Expression of pro-Cathespin L, which is responsible for the degradation of CD74, was highly expressed in fully activated microglia, but not in resting or mildly active microglia. Therefore, in future studies, we will determine if increased CD74 in NFTs is due to a dysregulation of Cathsepin L [12].

NFTs are a hallmark characteristic of AD pathology and are a result of tau accumulation. Oxidative stress and activation of cell cycle regulators are associated with NFT formation [22,23]. Tau and neurofilament proteins contain a high content of lysine-serine-proline (KSP) domains, which makes them uniquely adapted to oxidative attack. Oxidative stress through the activation of the MAP kinase pathways leads to tau phosphorylation [24]. CD74 also increases in response to oxidation and when stimulated by MIF induces phosphorylation of ERK1/2 [8].

The cell cycle hypothesis of AD states that cell cycle disruptions occur in AD and that activation of cell cycle regulators precedes the formation of NFTs [25]. Furthermore, markers from each phase of the cell cycle have been accounted for in degenerating neurons, which has led some to hypothesize that cells may re-enter the cell cycle through to the S phase, but for unknown reasons abort somatic division leading to degeneration of the neuron. CD74 regulates cell entry to the S phase in B cells. Cyclin E is expressed on initiation of the S-phase and is upregulated following CD74 stimulation [21]. Whether there is a connection between CD74 activation and disruption of the cell cycle in degenerating neurons is unknown, but may be an indication as to why CD74 is found in NFTs. In conclusion, CD74 is increased in microglia and neurons in AD patients. NFTs had the greatest increase in CD74, while microglia CD74 was only minimally increased. Our results are the first to find an increase of CD74 in NFTs. The mechanism and importance of CD74 in neurons has yet to be elucidated and further studies need to be conducted.

## Methods

### Immunocytochemistry

Postmortem hippocampal tissue from six confirmed AD patients (ages 87–67) and six aged matched control patients (ages 86–43) were fixed in methacarn (methanol-chloroform-acetic acid; 6:3:1) overnight at 4°C, dehydrated in ascending ethanol and embedded in paraffin. Six micron sections were cut and placed on plus coated slides (Fisher Scientific, 25 × 75 × 1.0 mm). Sections were then deparaffinized in xylene and hydrated through decending ethanol. Endogenous peroxidase was quenched with 3% hydrogen peroxide/methanol. Non-specific binding sites were blocked with 10% normal goat serum in TBS (150 mM Tris-buffered Saline, pH 7.6) followed by incubation in the primary antibody, CD74 (1:100, Santa Cruz). The sections were stained with the peroxidase anti-peroxidase method [26] with 3,3-diaminobenzidine (DAB) as the co-substrate as previously described.

### Immunoblotting

Poly-acrylamide gel electrophoresis was performed on hippocampal and frontal cortex tissue from four AD and four control cases. Samples were homogenized in TBS and 10 μg of protein was loaded per lane in Kelly's sample buffer. Proteins were resolved on 10% SDS-PAGE gels and transferred to Immobilon-P, PVDF membrane (Millipore Corp., Billerica, MA). Membrane was probed with CD74 after non-specific binding sites were blocked with 10% non-fat milk in TBS, followed by HRP linked secondary (ICN). Proteins were detected with immobilon western chemiluminescent HRP substrate (Millipore Corp., Billerica, MA). The membrane was stripped with stripping reagent and re-probed with actin as an internal loading control.

### Statistical Analysis

Densitometric analysis was performed with UN-SCAN-IT gel 6.1 software (Silk Scientific; Oren, UT). Significance was determined with the student t-Test.

## List of abbreviations

AD: Alzheimer disease; Aβ: amyloid-β; DAB: 3,3-diaminobenzidine; ERK: extracellular-regulated-kinase; IL-1β: interleukin-1β; IL-6: interleukin-6; KSP: lysine-serine-proline; MIF: macrophage migration inhibitory factor; MHC: major histocompatibility complex; MMSE: mini-mental state exam; NFT: neurofibrillary tangles; PGE_2_: prostaglandin E_2_; TBS: Tris-buffered saline, TNFα: tumor necrosis factor α.

## Competing interests

The authors declare that they have no competing interests.

## Authors' contributions

KJB and PLH carried out the immunocytochemistry; PLH, RJC, and XWZ participated in data analysis and interpretation; and KJB, XWZ, GP, and MAS drafted the manuscript. KJB, MAS and GC conceived of the study and participated in the design of the study. All authors read and approved the final manuscript.
